# Engineered models of the human heart: Directions and challenges

**DOI:** 10.1016/j.stemcr.2020.11.013

**Published:** 2020-12-18

**Authors:** Jeroen M. Stein, Christine L. Mummery, Milena Bellin

**Affiliations:** 1 Department of Anatomy and Embryology, Leiden University Medical Center, Leiden 2333ZA, the Netherlands; 2 Department of Applied Stem Cell Technologies, University of Twente, Enschede 7500AE, the Netherlands; 3 Department of Biology, University of Padua, Padua 35131, Italy; 4 Veneto Institute of Molecular Medicine, Padua 35129, Italy

**Keywords:** engineered heart tissue, human pluripotent stem cells, heart-on-a-chip, cardiovascular disease modeling, force of contraction

## Abstract

Human heart (patho)physiology is now widely studied using human pluripotent stem cells, but the immaturity of derivative cardiomyocytes has largely limited disease modeling to conditions associated with mutations in cardiac ion channel genes. Recent advances in tissue engineering and organoids have, however, created new opportunities to study diseases beyond “channelopathies.” These synthetic cardiac structures allow quantitative measurement of contraction, force, and other biophysical parameters in three-dimensional configurations, in which the cardiomyocytes in addition become more mature. Multiple cardiac-relevant cell types are also often combined to form organized cardiac tissue mimetic constructs, where cell-cell, cell-extracellular matrix, and paracrine interactions can be mimicked. In this review, we provide an overview of some of the most promising technologies being implemented specifically in personalized heart-on-a-chip models and explore their applications, drawbacks, and potential for future development.

## Teaser

In this review, M. Bellin and colleagues provide an overview of cutting-edge cardiac microphysiological systems as well as their strengths, drawbacks, and applications in disease modeling and cardiac toxicity testing. They describe how the multicellular and dynamic environment of the myocardium can be mimicked using microfabrication tools.

## Main text

### Introduction

Cardiovascular disease (CVD) accounts for 4.1 million deaths annually in Europe, and its global economic impact is estimated to be €950 million in 2030 (Timmis et al., 2020). Benefits from new cardiovascular drugs are not expected in the short term because of the paucity of biological models to identify biological targets for the human heart, rising costs in the pharmaceutical drug development pipeline, and failures in clinical trials due to unpredictable cardiac toxicity (Eder et al., 2016). One underlying reason for this is the pathophysiology of many cardiac diseases and drug effects on the healthy heart that are not captured well in rodents. Not only do ion channel expression and use differ between humans and rodents, but also variants and mutations causing or predisposing to CVD in humans may have little or no effect in transgenic mice even if there is a genetic equivalent. In addition, inflammation can modulate disease advance or recovery, and inflammatory cells in humans and mice also differ. Furthermore, it has been estimated that 70% of cardiac toxicity from clinical trials could be detected if preclinical screening methods were improved (Eder et al., 2016). There is thus a strong case for robust and, as necessary, more complex models of the heart based on human cells in drug discovery.

Several biological and technological advances have suggested how appropriate models for the human heart might be built. Specifically, human induced pluripotent stem cells (hiPSCs) derived from patients can now be used to produce different cell types of the heart using robust, chemically defined protocols (Giacomelli et al, 2017a, 2020; Passier et al., 2016). This can be combined with precise gene editing, for example, using CRISPR-Cas technology (Giacomelli et al., 2017a; Passier et al., 2016), to generate transgenic reporter cell lines for different lineages and introduce or repair CVD-relevant mutations to create isogenically matched pairs of diseased and healthy lines. There is now a wide range of patient hiPSC lines available bearing genetic mutations for channelopathies and cardiomyopathies (Giacomelli et al., 2017b). There are also many transgenic human embryonic stem cell (hESC) cardiac gene reporter and disease mutation-bearing lines, but these are ethically less acceptable in some countries. hiPSCs and hESCs (collectively called hPSCs) share many functional features and cardiomyogenic potential. Despite growing evidence of their utility, one major shortcoming has remained, namely, the immaturity of the hPSC-derived cardiomyocytes (hPSC-CMs). Solutions to this have been sought in another field developing in parallel: the application of two- and three-dimensional (2- and 3D) tissue engineering techniques to stem cell biology. Emerging models include several for the heart: cardiac microtissues (Giacomelli et al., 2017c), engineered myocardium (Eder et al., 2016), and heart-on-a-chip (HoC) devices (Zhang et al., 2015), in many cases with relevant sensors and environmental stimuli, such as mechanical and electrical stressors. In several of these formats, CMs appear to become more mature, especially when subjected to cyclic stress or in contact with non-CM cells of the heart (Abilez et al., 2018; Giacomelli et al., 2020; Ronaldson-Bouchard et al., 2018).

It is thus becoming possible to move beyond channelopathies to study multiple types of heart failure characterized by loss of contractile function. Variations and combinations of manufacturing methods allow HoCs to be customized for many different purposes. For instance, spatial organization of individual cell types with separated media compartments under fluidic flow can mimic the microscale geometry of the myocardium and enable conditional exposure to biochemical compounds (Maoz et al., 2017). Furthermore, electrical (Zhang et al., 2018b) or optical pacing (Lemme et al., 2020) can be implemented for wave propagation, cyclic stretch applied for mechanical loading (Parsa et al., 2017), and sensing systems introduced for functional assessment of CM contraction, electrical action potential, calcium transients, and stress responses (van Meer et al., 2016). Studies on engineered heart tissues (EHTs) (in which cardiac cells are embedded in an extracellular matrix [ECM] and self-organize into 3D myocardial structures around fixed anchor points) have shown that anisotropic (or directional) mechanical restrictions of CM contractility can enhance the structural, electrophysiological, and metabolic maturity of hPSC-CMs (Lemoine et al., 2017; Nunes et al., 2013; Ronaldson-Bouchard et al., 2018; Tiburcy et al., 2017), creating an avatar for the (postnatal) human myocardium.

Aside from providing more holistic insights into the mechanisms of genetic or acquired diseases, drug responses, or cardiac toxicity, these models are increasingly used by academia and pharmaceutical companies as (high-throughput) methods of screening new compounds to counteract arrhythmias, fibrosis, or other disabling heart diseases. Here, we provide an overview of the most promising microphysiological systems and HoC models for studying the human heart.

### Reverse engineering heart physiology

**Figure 1:**
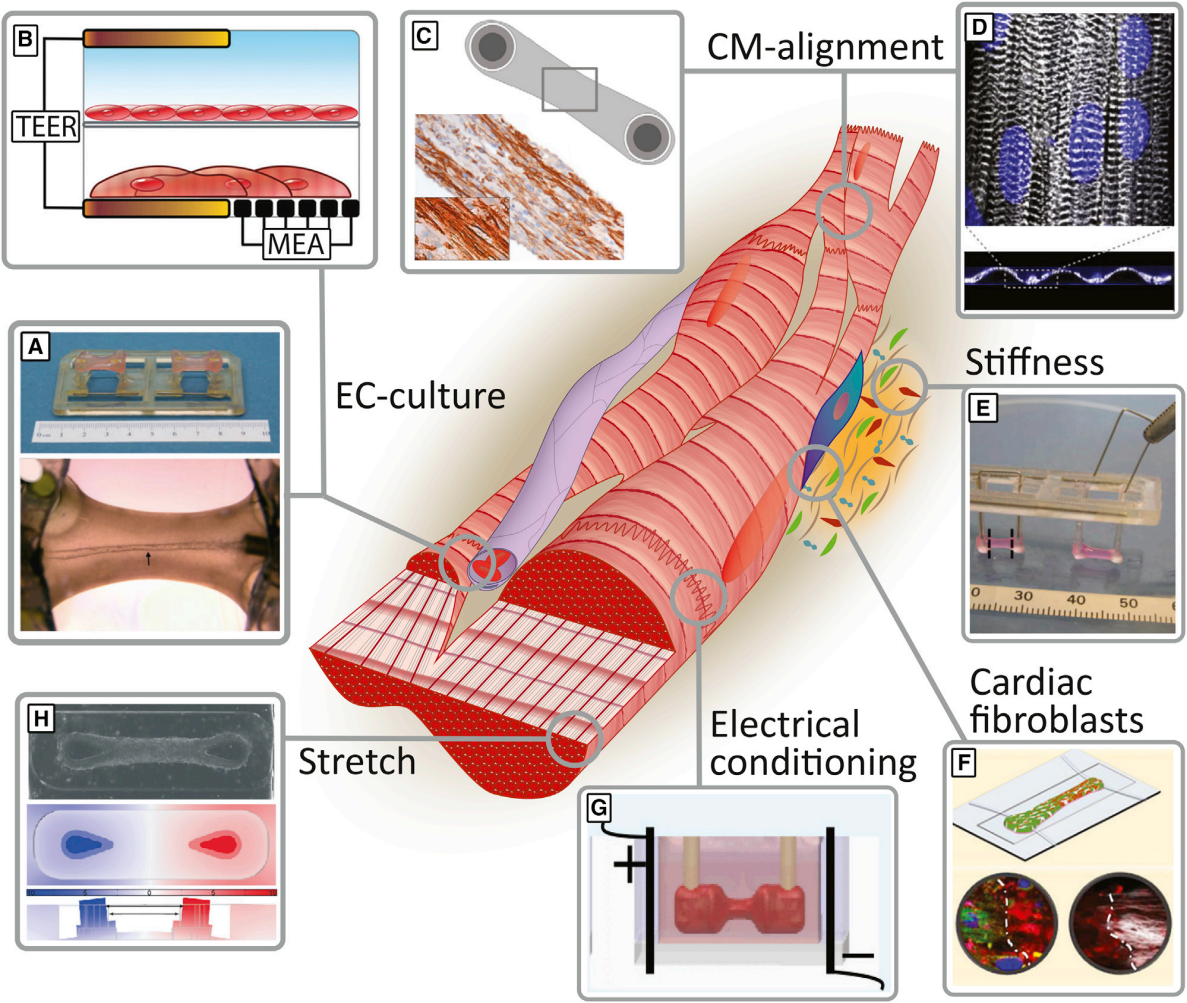
Methods for Mimicking *In Vivo* Heart Physiology Using Organ-on-a-Chip Technology Models to increase cardiac tissue mimicry and cardiomyocyte maturation compared with standard 2D cultures are shown. There are many ways of co-culturing cardiomyocytes, endothelial cells, and cardiac fibroblasts in a heart-on-a-chip, via either (A) bioengineering a tube within a 3D tissue (Vollert et al., 2013) or (B) engineering a dual-compartment chip (Maoz et al., 2017). Alignment of cardiomyocytes in an HoC model was achieved in both (C) 3D tissues (Hirt et al., 2014) and (D) 2D monolayers (Lind et al., 2017) to reach higher levels of maturity. (E) An increase in stiffness, which is a hallmark of adverse remodeling in diseased myocardium, was achieved by inserting iron rods into pillars (Hirt et al., 2012). (F) A focal myocardial scar (white dash-dotted line) was created by incorporating regions with high and low percentages of cardiac fibroblasts within one tissue (Wang et al., 2019). In the heart, (G) electrical stimulation (Ronaldson-Bouchard et al., 2019) and (H) mechanical stretch (Parsa et al., 2017) are beneficial for reflecting the physiological cardiac tissue environment. CM, cardiomyocyte; EC, endothelial cell; MEA, multielectrode array; TEER, transepithelial electrical resistance. (A–H) were reprinted with authorization from the copyright owner. For further permissions the reader is directed to the corresponding publishers.

Organs on chips are designed to re-create the smallest functional unit of an organ. The intention is that biological, spatial, and mechanical stimuli can be customized to recapitulate the actual tissue *in vivo*. Identifying minimal organ functionalities is crucial for reverse engineering organ physiology. For blood vessels, this would be fluid flow; for the intestine, peristalsis; for the lung, airflow and cyclic contraction. The heart is a complex organ for which not only different cell types and biophysical properties should be taken into account, but also CM anisotropy (directional alignment), fluid flow through blood vessels, cyclic stretch, and ECM interactions (Figure 1).

Excitation of the heart starts in the sinoatrial node, spreads through the atria to the atrioventricular node, and then via the His bundle and Purkinje fibers to the ventricle, initiating the contraction of each individual cell and the whole tissue. In HoC models, integrated electrodes can provide this stimulus, and many studies have already shown that chronic alternating electrical stimulation increases maturation in hiPSC-CMs (Hirt et al., 2014; Ronaldson-Bouchard et al., 2018). A study where engineered skeletal muscle was built showed that light-induced channel Rhodopsin in motor neurons could stimulate contraction in nearby 3D-muscle tissue-on-a-chip (Uzel et al., 2016). The heart is continuously exposed to mechanical load. Mimicking cyclic stretch *in vitro* has a large impact on the maturity and contractility of hiPSC-CMs (LaBarge et al., 2019). In 2D monolayers, uniaxial stretch has enabled identification of responses to mechanical stimuli in diseased hiPSC-CMs from cardiomyopathies (Martewicz et al., 2019). In 3D models, mechanical loading of hPSC-CM bundles improved sarcomere alignment and length, twitch force, and expression of structural proteins (Zhang et al., 2017).

Moreover, the organization and alignment of the ECM influence CM function in health and disease. Several HoCs are designed to control CM alignment using geometric cell-adhesion coatings or physical constraints (Martewicz et al., 2019; Nunes et al., 2013). Micropatterning ridges or protein substrates on culture vessels have been shown to benefit CM function and maturity and cardiomyogenic differentiation potential (McCain et al., 2013). This was clearly reflected in a relevant disease phenotype observed in a model of Barth syndrome using TAZ mutant patient lines (Wang et al., 2014) and drug responses (Chang et al., 2018).

### Cell sources

It has become increasingly clear that incorporation of other cell types is crucial for proper CM function and therefore complex physiological modeling (Wang et al., 2018). Many 3D models rely on self-assembly of a hydrogel mixed with CMs and fibroblasts (Breckwoldt et al., 2017; Tiburcy et al., 2017) or endothelial cells (ECs) (Giacomelli et al., 2017c). However, when modeling more complex diseases, other cell types, e.g., immune, epicardial, nodal, nerve, or blood cells, might be necessary (Wang et al., 2018).

Methods for producing, culturing, and differentiating hPSCs have increasingly improved in cost, effort, and efficiency over the past 12 years (Devalla and Passier, 2018). The many methods for differentiating CM subtypes (atrial, ventricular, and nodal) from hiPSCs are discussed elsewhere (Mummery et al., 2012; Protze et al., 2019).

As mentioned earlier, standard monolayer differentiations give rise to CMs with an immature phenotype, reflected in cell morphology, disorganized sarcomere arrangement, low electrical action potentials, fetal-like metabolism and energy use, mononucleation, and low mitochondrial volume. Together, these result in low contractile properties and physiological phenotypes that do not resemble adult CMs (Yang et al., 2014). HoC technology in principle allows environmental factors to be controlled, such as mechanical or electrical conditioning and geometric constraints, as well as incorporation of different cell types; this can promote CM maturation in addition to mimicking cardiac tissue organization. Moreover, microfabrication enables spatial definition of subsections of the ventricle and atrium of the heart (Zhao et al., 2019). Cardiac fibroblasts (CFs) are involved in fibrosis, but they are being recognized as playing a role in many other cardiac diseases (Dostal et al., 2015). CFs are indeed essential for the electrical conductivity of the myocardium and secrete cytokines and produce ECM, which are necessary for CM function (Dostal et al., 2015). Because of their different developmental origins, CFs in the human heart are a heterogeneous population of cells (Moore-Morris et al., 2016; Tallquist and Molkentin, 2017). The majority arise from the epicardium, the epithelial layer covering the heart surface, but other sources include neural crest cells, the vascular system, or circulating progenitors (Dostal et al., 2015; Protze et al., 2019). This complexity has made it difficult to isolate and classify CFs based on their gene expression profile or membrane marker expression. This, in turn, challenged the field to induce differentiation of CFs *in vitro* from hiPSCs. Several protocols have now been established via lineage specification of either second heart field progenitors (Zhang et al., 2018a) or epicardial progenitors (Giacomelli et al., 2020; Guadix et al., 2017; Zhang et al., 2019).

ECs constitute the largest population of non-CMs in the adult human heart, with a microvessel next to every CM (Pinto et al., 2016). *In vitro*, ECs can increase hiPSC-CM differentiation efficiency (Pasquier et al., 2017) and maturation (Giacomelli et al., 2017c; Vuorenpää et al., 2017). Incorporation of an EC “barrier” to mimic the blood vessel wall in HoC models is now a broad area of research (reviewed by Cochrane et al., 2018). Inclusion of vasculature in HoC enables the addition of physiological factors like circulating red blood cells (to mimic thrombosis if there is a constriction in the microchannel; Westein et al., 2013), immune cells, reperfusion injury, or transient addition of relevant compounds.

ECs can be differentiated from hPSCs through lineage specification by the addition of small molecules or via the expression of the core endothelial transcription factors ETV2 and GATA2 (Cochrane et al., 2018). However, ECs exhibit plasticity and differ in function and gene expression profiles between different organs. For example, cardiac microvascular endothelium expresses several cardiac-specific secreted factors, like Endothelin-1 and Neuregulin, which support CM survival (Drawnel et al., 2013; Lemmens et al., 2006). hiPSC-ECs differentiated through a cardiac progenitor intermediate similarly express cardiac-related genes. Cardiac ECs themselves are in turn supported by smooth muscle cells and pericytes, which are known to enhance barrier function and inflammatory responses (Cochrane et al., 2018; Halaidych et al., 2018). These can also be differentiated from hPSCs (Orlova et al., 2014; Protze et al., 2019).

### Readouts

One of the main features of organ-on-a-chip (OoC) technology is the ability to integrate sensors, which enables researchers to collect direct and quantitative outputs of cell behavior. This is especially important for electrically and mechanically active cells like CMs where non-disruptive measurement of function is essential.

Microscopy in various forms is widely used to assess cell morphology, behavior, and distribution. Most chips are designed to enable microscopic imaging with a short objective-sample distance. When an optical window is incorporated into an HoC, for example, it is possible to use a range of voltage- or calcium-sensitive dyes or genetically encoded calcium/voltage indicators to detect responses, or sarcomere reporters to provide insights into CM physiology, drug responses, and disease phenotypes.

The most straightforward readouts for CMs, however, are the contractile properties, which can be assessed either as relative pixel displacement or as absolute force of contraction (FOC). Relative pixel displacement relies on computing contractile kinetics of any tissue by edge detection or frame similarity (van Meer et al., 2016). This method does not need specialized hardware and is therefore widely applicable. Absolute force measurements rely on chip systems where the CMs are attached to and displace a material with known stiffness (van Meer et al., 2016) (see below). Alternatively, traction-force microscopy can be used, although this is typically applicable to monolayer cultures.

Multielectrode arrays (MEAs) are used in both academic and pharmaceutical research to measure the electrical field potential of cardiac cells (Tertoolen et al., 2018). Modern microfabrication methods enable implementation of electrodes in complex devices. For instance, Maoz and colleagues elegantly combined an MEA with impedance measurements in different cell compartments on a microfluidic chip to determine field potentials of the CMs and transepithelial membrane resistance measurements of an EC barrier simultaneously (Figure 1B; Maoz et al., 2017). The mechanical strain of hiPSC-CMs in a monolayer can also be measured in HoCs. Although this increases fabrication costs and time, data acquisition is greatly accelerated compared with optical imaging methods (Lind et al., 2017).

### 2D devices

2D-CM cultures are often regarded as inadequate for characterization of cardiac physiology, due to less organized cell-cell interactions, lower maturation status, and unexpected responses to some drugs (Mittal et al., 2019). However, 2D cultures have certain advantages, notably ease of use and high throughput, which make them the preferred format for preclinical testing and large-scale pharmaceutical drug screens. As stated above, though, many of the drawbacks can be circumvented in several ways, one being topological patterning of the culture substrate (Figure 1D). Sarcomeric organization (Salick et al., 2014), isoproterenol-mediated PLN and TNNI phosphorylation (Jung et al., 2016), and hiPSC-CM differentiation efficiency (Xu et al., 2017) have all been shown to increase when hiPSC-CMs were guided to alignment by their culture surface.

Another major advantage of 2D-patterned substrates is the ability to distort the connexin/gap junction-mediated propagation wave. The development of a patterned muscular thin film has enabled Park’s group to study arrhythmia in an hiPSC model for catecholaminergic polymorphic ventricular tachycardia under high-exercise conditions (Park et al., 2019). Re-entry was induced by isoproterenol and channel rhodopsin-driven pacing frequencies of up to 3 Hz and imaged by calcium fluorescence. Inhibiting CaMKII-mediated RYR2 phosphorylation abrogated exercise-induced arrhythmias.

### 3D devices

Modern techniques enable a multiplicity of possibilities for combining microfabrication to guide 3D cell positioning and compartmentalization with integrated sensors and enabling microfluidic flow. However, one design that has demonstrated advanced maturation of cardiac tissue, as well as direct contraction measurements, involves 3D self-assembly of a single cardiac muscle strand around microfabricated anchor points (Figure 1C). These EHTs increase physiological mimicry significantly by providing mechanical resistance to the cardiac tissue through anchoring and consequently maturity status, but with a concomitant decrease in spatial control. This approach is now generally divided into two categories: hanging pillars and standing pillars.

Hanging pillars were pioneered over a decade ago by Eschenhagen’s group (Hansen et al., 2010), using neonatal rat CMs and fibrin gel. Since then, there have been major improvements in EHT technology, including automated analysis of pillar deflection (Stoehr et al., 2014), chronic electrical stimulation (Hirt et al., 2014), and integration of hiPSC-CMs (Breckwoldt et al., 2017). Bench-to-bedside analysis of hiPSC-CMs derived from a family affected by hypertrophic cardiomyopathy (HCM) in EHTs recapitulated several disease-specific traits, such as hypertrophy, altered calcium response, hypercontractility, and sarcomeric disarray (Prondzynski et al., 2019). After the efficacy of L-type calcium-channel inhibition with Diltiazem was proven in the EHTs, the drug was administered to HCM-affected family members, improving their electrical phenotype. This research showcases the potential of combining HoC research and CRISPR-Cas gene editing in hiPSCs and may pave the way for improved personalized or precision medicine.

A more detailed analysis of CM maturation in EHTs was performed by Ronaldson-Bouchard and co-workers, who redesigned the hanging pillar model to improve alignment and centralization of force through the middle of the tissue (Figure 1G) (Ronaldson-Bouchard et al., 2019). Using a pacing protocol of increasing frequencies up to 6 Hz, maturation was claimed in terms of ultrastructural morphology, calcium handling, and gene expression (Ronaldson-Bouchard et al., 2018), although this was not completely paralleled by a similar level of maturation in metabolism and electrophysiology. However, after publication, the authors provided a “major correction” to this paper (April 2019), and subsequent work by a partially overlapping group of authors apparently failed to achieve CM maturation using similar pacing protocols, albeit in the BioWire system (Zhao et al., 2019).

Standing pillars offer some advantages and disadvantages. The main advantage is the decrease in the components necessary and consequentially costs, handling, and contamination risk. Tiburcy and colleagues have miniaturized their model to fit in a 96-well plate, enabling increased throughput for compound screening (Mills et al., 2019). However, in using standard microfabrication techniques it remains difficult to regulate tissue height on the pillar via design alterations. This affects force analysis, tissue pre-load, tissue formation, cellular behavior, and variability in inter-hiPSC-line comparisons when experiments are performed at different times. Dostanic and colleagues have further miniaturized the standing pillar design and performed extensive mechanical characterization of the pillars (Dostanic et al., 2020).

Aside from the muscle strands around pillars, this self-assembly method with direct force readout allowed formation of cardiac tissues between wires (Zhao et al., 2019). These muscle strands, termed BioWires, were made from atrial and ventricular hiPSC-CMs, showing distinct reactions to chamber-specific drugs. Moreover, this chip did not retain fluorescent hydrophobic compounds, which could make high-throughput screening methods on 3D cardiac tissues more reliable.

HoC tailored designs have been adapted to mimic increased systolic ventricular pressure in the cardiac tissues, relevant for heart disease modeling. Hirt and co-workers extended the hanging pillar design with stiff iron rods inserted into the pillars (Figure 1E). This resulted in a hypertrophic response in the EHTs, with reduced contractile force and relaxation velocity, fibrosis, and reactivation of fetal genes. Furthermore, inhibiting certain pathways with microRNAs attenuated this hypertrophic phenotype and improved EHT function (Hirt et al., 2015). A dynamic method of inducing functionality is growing tissues on pillars standing on a flexible membrane, which enables pneumatic stretching of the tissue (Figure 1H) (Parsa et al., 2017).

To date, there have been few studies integrating sensors in this type of 3D device. The optical tracking of pillars or wires is inexpensive and robust across laboratories, but much information could be missed by looking only at contraction dynamics. When a calcium-sensitive fluorophore was used in hanging pillar-type EHTs, positive calcium-frequency and force-frequency relationships up to 2.5 Hz were registered (Saleem et al., 2020), hallmarks of CM maturity.

The non-CM cell types used in these 3D cultures are typically primary human fibroblasts, isolated from skin (Cyganek et al., 2018) or the heart (Parsa et al., 2017; Zhao et al., 2019). Interestingly, Tiburcy and co-workers created tissues with and without human foreskin fibroblasts. The fibroblasts were essential for hydrogel condensation, with 70/30 hiPSC-CM/fibroblast ratios optimal for FOC (Tiburcy et al., 2017). The best differentiation protocols yield CM population purities >95%, which have been shown to be capable of forming EHTs without additional fibroblasts in some studies, although just a few residual non-CM cells might contribute to tissue formation and organization (Mills et al., 2017). Multiple studies have in fact shown that even without intentional inclusion of non-CMs, CD31^+^ ECs and CD90^+^ stromal cells can still be detected in the tissue (Abilez et al., 2018; Mills et al., 2017). These may derive from intermediate or residual cardiac progenitors that arise during differentiation.

### Outlook

The HoC field has rapidly evolved over the past decade and is providing a wide range of tools for fundamental research, high-throughput compound screening, and possibly precision medicine. Several technological advancements have mediated this progress: first, hPSC technology, which allows the creation of disease-specific HoCs, paving the way for patient (group)-specific OoC systems. The development of cardiac cell differentiation protocols that now go beyond CMs to a range of essential non-CM cell types enables researchers to pinpoint cell-specific contributions to heart physiology and disease. Second, advances in microfabrication techniques offer many combinations of geometrical designs and integration of “environmental” factors, mechanical stressors, and electrical and biochemical sensors to tailor OoC models to end-user requirements.

As the OoC field continues to renew and devices become more refined, many more innovative chip designs can be expected that will advance to the stage of independent validation. This will increase end-user confidence in the relevance of HoC outcomes and conclusions. However, there is also movement toward improving existing designs and solving complex problems. For example, the incorporation of fluid flow in channels lined with ECs mimicking blood vessels but also containing macrophages could simulate inflammation that underlies many cardiac diseases or indentations that simulate atherosclerotic plaques.

There is also new focus on measuring absolute FOC in HoCs, which should facilitate standardization across different laboratories, benchmarking of new HoC devices, and cross-comparison of different models. However, normalization to the cross-sectional diameter of the tissue or the number and viability of contractile cells in the tissue will be necessary. This is crucial for encouraging implementation of HoCs in drug-development pipelines. As mechanical and electrical stimulation enhanced tissue maturation, these modules could be of increasing interest as physiological models and for pharmaceutical screening.

**Figure 2:**
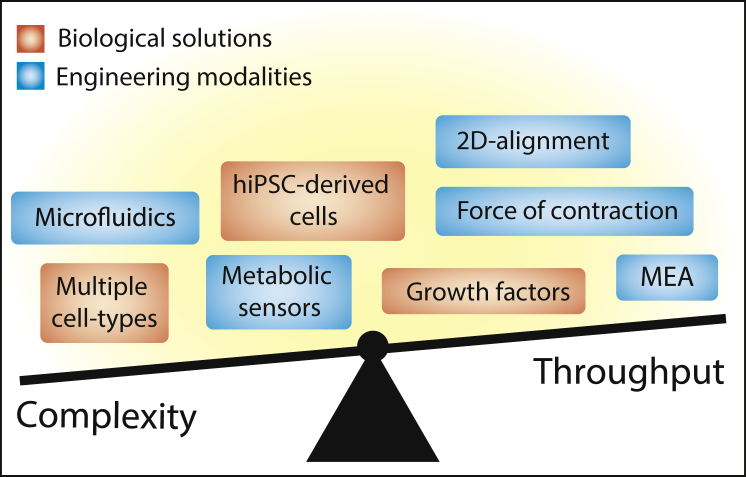
The Trade-Off between Complexity and Throughput in Heart-on-a-Chip Research In most cases, more complex models are less cost and time efficient. Complex problems require complex solutions, although increased optimization time is often unavoidable. hiPSC, human induced pluripotent stem cell; MEA, multielectrode array.

To further enhance the potential of HoC technology, the inclusion of additional optional modules could be of value: electrodes, sensors, and mechanical stimulation could all in principle be added to any design with the present microfabrication tools. A concomitant increase in optimization and assay times and fabrication costs would be unavoidable (Figure 2), but if mechanical and electrical stimulation enhanced tissue maturation, these modules would nonetheless be of high interest for both physiological models and pharmaceutical screening. The rise in commercialization of OoC platforms will ultimately reduce fabrication costs, which in turn will help research groups adopting new models to add their expertise.

## Author contributions

Conceptualization, J.S., C.M., and M.B.; Investigation, J.S.; Data Curation, J.S.; Writing – Original Draft, J.S.; Writing – Review & Editing, C.M. and M.B.; Visualization, J.S.; Supervision, C.M. and M.B.; Funding Acquisition, C.M. and M.B.

## Conflict of interest

C.M. is a co-founder of Ncardia BV.
